# The Immune Interplay between the Host and the Pathogen in *Aspergillus fumigatus* Lung Infection

**DOI:** 10.1155/2013/693023

**Published:** 2013-07-30

**Authors:** Helioswilton Sales-Campos, Ludmilla Tonani, Cristina Ribeiro Barros Cardoso, Márcia Regina Von Zeska Kress

**Affiliations:** Departamento de Análises Clínicas, Toxicológicas e Bromatológicas, Faculdade de Ciências Farmacêuticas de Ribeirão Preto, Universidade de São Paulo, Avenida Do Café S/N, 14040-903 Ribeirão Preto, SP, Brazil

## Abstract

The interplay between *Aspergillus fumigatus* and the host immune response in lung infection has been subject of studies over the last years due to its importance in immunocompromised patients. The multifactorial virulence factors of *A. fumigatus* are related to the fungus biological characteristics, for example, structure, ability to grow and adapt to high temperatures and stress conditions, besides capability of evading the immune system and causing damage to the host. In this context, the fungus recognition by the host innate immunity occurs when the pathogen disrupts the natural and chemical barriers followed by the activation of acquired immunity. It seems clear that a Th1 response has a protective role, whereas Th2 reactions are often associated with higher fungal burden, and Th17 response is still controversial. Furthermore, a fine regulation of the effector immunity is required to avoid excessive tissue damage associated with fungal clearance, and this role could be attributed to regulatory T cells. Finally, in this work we reviewed the aspects involved in the complex interplay between the host immune response and the pathogen virulence factors, highlighting the immunological issues and the importance of its better understanding to the development of novel therapeutic approaches for invasive lung aspergillosis.

## 1. Introduction

An infection due to an *Aspergilli* was first described in animals in 1815 when its presence was observed in the air sacs and lungs of a crown [1]. However, the first human case was only described almost 30 years later in Scotland, when the sputum of a patient was microscopically analyzed by Benett [2]. Those findings were followed by the description of bronchial and pulmonary aspergillosis in humans by Virchow [3] and the remark that this infection may occur coupled to other lung diseases such as tuberculosis [4]. Although the disease was already known by that time, the fungus *Aspergillus fumigatus* was only described by Fresenius in 1850 when air sacs and bronchi of a great bustard were analyzed [5]. Therefore, aspergillosis was firstly described as an opportunistic infection when an immunocompromised patient had a disseminated mycosis with the presence of *A. fumigatus* in lung and kidney, besides *Candida* in liver and spleen, or the concomitant presence of both fungi in other tissues [6].

Ubiquitous in nature and without geographic predilection, *Aspergillus* species are found in the air, food, water, soil, and decomposing vegetation where they play an essential role in recycling environment carbon and nitrogen. Inhalation of *Aspergillus *spp. conidia by immunocompetent individuals rarely has any adverse effect, since the conidia are efficiently eliminated by immune mechanisms. Thus, the isolation of *A. fumigatus* from respiratory secretions in normal hosts generally reflects colonization rather than infection [7–9]. In the last decades, with the advent of solid organ and bone marrow transplantation, the increased use of immunosuppressive drugs, and the epidemic infection with the human immunodeficiency virus (HIV), the disease caused by *A. fumigatus* has emerged as a severe infection in immunocompromised patients. Established infection in these patient groups has proven difficult to eradicate, and despite significant advances in antifungal therapy in recent years, overall mortality with invasive disease remains high [10]. The most frequent pathogenic agent of aspergillosis is *A. fumigatus, *followed by *A. flavus *and* A. niger *[11]. Among them, the first has some characteristics which may allow a greater adhesion to the airways before invasion such as its ability to bind to laminin [12], thus conferring an evolutionary advantage to *A. fumigatus* during infection over the others.

The incidence of invasive aspergillosis is increasing [13–16], and *A. fumigatus* causes approximately 90% of this disease [8]. Thus, the development of pulmonary aspergillosis, which involves severe pulmonary manifestations, relies on a series of predisposing aspects and on a complex interplay between both the host immune competence and the pathogen virulence factors.

## 2. *Aspergillus fumigatus* Virulence Factors

The virulence of *Aspergillus fumigatus* is multifactorial and is combined with both the immune status of the patient and the biological characteristics of the fungus. There is a high connection between them, as demonstrated by the differences in the activation of the innate immune system which depend on the *Aspergillus* spp. morphology, growth stage, environment sensing and species, representing a key factor in fungal pathogenicity [17]. Once the fungus conidia reached the host environment, which is a different condition found in their normal environment niche, it must continually adapt to survive. These adjustments are classified according to the process they are involved in, for example, thermotolerance, toxins, cell wall composition and conservation, resistance to the immune response, nutrient uptake, signaling, metabolism, and response to stress condition.

The decaying organic matter is the normal environment for *A. fumigatus*, which is subjected to a wide temperature variation as a consequence of intense microorganism activity. The ability to grow at 37°C is shared by all successful human pathogens and is a feature that has been shown to correlate with virulence potential. *A. fumigatus* can grow between 37 and 50°C and is therefore more resistant and has better thermotolerance than other *Aspergillus* species [18, 19]. The ribosomal biogenesis proteins encoded by *crgA* [20], *α*-mannosyltransferase (*kre2/mnt1*) [21], and the endoplasmic reticulum-transmembrane sensor encoded by *ireA* [22] are to date the proteins associated with thermotolerance growth and hypovirulence, when the respective genes were deleted from *A. fumigatus *genome.

 The fungal cell wall of *A. fumigatus* represents the first point of contact with the hosts and plays an important role in the infection process. It is a polysaccharide-based three-dimensional network which is a physical protection and provides a dynamic structure that is continuously changing as a result of the modification of the culture conditions and environment stress. The cell wall is composed by *β*(1,3) and *β*(1,4)glucans, *α*(1,3)glucans, chitins, and galactomannans [23]. A melanin layer and sialic acid are found in the conidia surface, and a hydrophobic component layer is present on both conidia and hyphae [24]. The skeleton pattern arrangement of the cell wall is composed by the *β*(1,3)glucan branched with *β*(1,6)glucan that is covalently bound to chitin and *β*(1,3/1,4)glucan. Dectin-1, an innate immune receptor of the mammalian cell, recognizes the fungal cell wall pathogen-associated molecular patterns [25] and may initiate immune response against the fungus. Additionally, *β*-glucan is present in almost all fungi and has been used for the diagnosis of invasive mycosis [26].

The most abundant polysaccharide in the *A. fumigatus *cell wall is *α*(1,3)glucan and among the three *α*(1,3)glucan synthases identified in the fungus genome, only Δ*ags3 *mutant has shown virulence changes (hypervirulence) in an experimental mouse model of aspergillosis [27]. Seven chitin synthases encoding genes have been identified in *A. fumigatus *genome, among them four genes were assessed for virulence profile and only the *chsG* null mutant strain displayed hypovirulence phenotype [28]. The main exoantigens released by the fungus during tissue invasion are the galactomannans [9], which may activate the innate immune response away from the focus of the infection. In the cell wall the galactomannans are composed by a linear *α*-mannan backbone and short chains of *β*(1,5)galactofuranose residues [29]. Galactofuranose biosynthesis starts with the isomerization of UDP galactopyranose to UDP galactofuranose by UDP galactomutase encoded by the *glfA* gene. The absence of UDP galactomutase in *A. fumigatus *led to attenuated virulence in a mouse model of invasive aspergillosis [30].

Glycosyl phosphatidyl inositol (GPI) motif proteins anchored to the plasma membrane play important roles in the biosynthesis and organization of the fungal cell wall. Many proteins such as cell surface enzymes, receptors, and adhesion molecules are anchored to the cell membrane by the GPI anchor which in turn may transfer the cell-wall-related information across the cell membrane. In this context, the absence of glucanosyltransferase encoded by *gel2 *gene has been related to hypovirulence of *A. fumigatus* [31]. Another gene related to the virulence attenuation in *A. fumigatus *is *Afpig-a, *which encodes the catalytic subunit of a complex that catalyzes GPI anchor biosynthesis [32]. On the other hand, *ecm33* gene, which encodes a GPI-anchored protein, plays an important role in maintaining fungal cell wall integrity, and the absence of this enzyme enhances the virulence of the fungus [33].

Signal transduction pathways play a critical role in the biology of all living cells contributing to the integration of environmental cues into appropriate cellular activities. Proteins that participate in the signal transduction such as the G-proteins, MAP kinases, adenylate cyclases, and protein kinases (PKA) have been associated with virulence control and development of fungal pathogens [34]. The cyclic adenosine monophosphate (cAMP) PKA dependent is the central component of the cAMP signaling cascade. PKA is a serine/threonine kinase composed by the catalytic subunit *pkaC1* and the regulatory subunit *pkaR*. The central messenger of the signal transduction pathway is cAMP, produced by the adenylate cyclase, which is regulated by GpaB, a G-protein-*α* subunit. The malfunction of the PKA pathway by the disruption of *gpaB*, *pkaC1* [35], or *pkaR* [36] in *A. fumigatus *leads to virulence attenuation in mice. cAMP, the second messenger molecule, is produced after perception of an extracellular signal by the G-protein-coupled receptor (GPCR). GprC and GprD in *A. fumigatus *are GPCRs and important for fungal metabolism regulation, conidia germination, and hyphae elongation and branching. Furthermore, these receptors regulate resistance towards environmental stress caused by reactive oxygen intermediates and elevated temperatures. They also play a role during the infection process, as the mutant strains were attenuated in virulence. Additionally a connection of the receptors with calcineurin-mediated signal transduction [37] is proposed.

Calcium-dependent signaling mechanisms in fungi have been associated with the regulation of wide variety responses to stress including survival in the host environment and resistance to antifungal drugs [38]. Calcium enters the cell via plasma membrane channels in response to external stress and activates the calcium-binding protein calmodulin that in turn activates calcineurin, a protein phosphatase responsible for the stimulation of downstream target genes. In fungi calcineurin regulates localization and activity of Crz1p-like transcription factor [39, 40]. The deletion of the catalytic subunit of calcineurin, *calA/cnaA* [41, 42] and *crzA* [43, 44] in *A. fumigatus*, led to significant defects in conidial germination, polarized hypha growth, cell wall structure, and attenuation of mortality rate in a neutropenic murine model of invasive pulmonary aspergillosis. Calcineurin and Ras proteins have been implicated in parallel activity in the regulation of hyphal and cell wall formation [45]. Ras are small monomeric GTPases proteins that act as molecular switcher which transduce signals from outside of the cell to the signal pathways inside the cell. Three Ras proteins have been studied in *A. fumigatus*, RasA, RasB, and RhbA. RasA is critical for polarized morphogenesis and cell wall stability [46] and RasB have been implicated in the control signaling modules important to the directional growth of fungal hyphae [47]. On the other hand, RhbA is implicated on nitrogen-dependent nutrient sensing and acquisition [48]. The deletion of Ras proteins encoding genes, *ΔrasA, ΔrasB*, and *ΔrhbA* leads to *A. fumigatus* virulence decrease in murine model of invasive pulmonary aspergillosis [46–48]. 

The genome of *A. fumigatus *conidia is protected from enzymatic lysis, ultraviolet light and oxidation by a gray-green melanin layer adhered to the cell wall [49]. The transcription of the essential genes for the biosynthesis of both types of melanins, pyomelanin and dihydroxinaphthalene (DHN)-melanin, are detected during infection and also protect *A. fumigatus *against reactive oxygen species (ROS), which are important compounds from the host innate immunity against pathogens [50, 51]. Pyomelanin production is associated with the tyrosine degradation pathway by the oxidative polymerization of an intermediate of the pathway, the homogentisic acid (HGA) [50]. In response to human neutrophils [52] and dendritic cells [53], four genes of the tyrosine degradation pathway (*hppD, hmgX, hmgA, *and *fahA*) showed increased transcription, leading to believe that pyomelanin is involved in fungal survival by escaping from the host immune system. DHN-melanin production starts by the polyketide synthase Alb1/PksP. The presence of a functional *alb1/pksP* gene in *A. fumigatus* conidia is associated with an inhibition of phagolysosome fusion in human monocyte-derived macrophages [54], which can justify the virulence attenuation of the pigmentless *alb1/pksP* null mutant in murine infection model [55–57]. 

Macrophages and polymorphonuclear cells such as neutrophils produce reactive oxygen species (ROS) as defense mechanism against the fungal conidia and hyphae, respectively. On the other side, the fungus produces specific enzymes for ROS detoxification. One category of ROS detoxification proteins is the catalases peroxidases, Cat1/CatB and Cat2/KatG, produced by the fungus mycelia. The null mutant of each gene in *A. fumigatus* led to mycelial hydrogen peroxide sensitivity and virulence reduction in the lungs of immunosuppressed rats [58]. Additionally, the cyclic AMP-dependent protein kinase (PKA), a well-known regulator of stress response in eukaryotes, contributes to the growth, germination, ROS response, and the virulence response of *A. fumigatus* [36]. Another group of genes related to oxidative stress response are the fatty acid oxygenases *ppoA, ppoB*, and* ppoC*, which are similar in sequence to specific mammalian prostaglandin synthases, the cyclooxygenases. The fatty acid oxygenase encoding genes in *A. fumigatus* were tested for virulence, and the triple mutant strain was found to be hypervirulent in an invasive murine model, showing increased tolerance to hydrogen peroxide [59]. Finally, the null mutant of a nonribosomal peptide synthetase gene, *pes1* showed a significant reduction in virulence in the *Galleria mellonella* model system and an increased sensitivity to oxidative stress in culture and during neutrophil-mediated phagocytosis [60].

 In fungi, such as *A. fumigatus*, transcription factors represent an important genetic component for the establishment of an infection by the activation or repression of different mechanisms that regulate virulence and pathogenicity. The putative C_2_H_2_ transcription factor DvrA was identified in *A. fumigatus* as a negative regulator of host cell damage and stimulation as well as virulence during invasive pulmonary disease. The deletion of this gene led to the stimulation of CCL20, interleukin-8, and the tumor necrosis factor mRNA expression in a pulmonary epithelial cell line. Additionally, there were increased virulence and pulmonary inflammatory response in neutropenic mouse model of invasive pulmonary aspergillosis [61]. In a similar way, the deletion of the transcription factor Ace2 in *A. fumigatus *induced accelerated mortality, greater pulmonary fungal burden, and increased pulmonary inflammatory responses in nonneutropenic mice immunosuppressed with cortisone acetate. The phenotype of *Δace2* mutant in *A. fumigatus* was characterized by dysmorphic conidiophores, reduced conidia production, and abnormal conidial cell wall architecture, besides the reduced mRNA expression of* ppoC*, *ecm33,* and *ags3*. The null mutants of these genes have shown increased virulence in mice, as well as other phenotypic similarities to the *Δace2* mutant [62].

The filamentous fungi are well known microorganisms producers of secondary metabolites [63]. It is believed that the production of these secondary metabolites is linked to the protection of the fungus against the host [64]. In fungi, the genes required for the biosynthesis of secondary metabolites are clustered [65]. The gene *laeA* encodes the transcription factor methyltransferase which is the global regulator of the secondary metabolite clusters in *A. fumigatus* [66, 67]. The deletion of *laeA* in this fungus reduces the secondary metabolite production, including gliotoxin and reduces the virulence in a murine neutropenic model [68]. Gliotoxin is a member of the epipolythiodioxopiperazine class of toxins and is both the major and the most potent toxin produced by *A. fumigatus*. This fungus maintains its normal virulence after deletion of the gene that encodes a nonribosomal peptide synthase of the gliotoxin biosynthetic cluster,* gliP*, in neutropenic mice immunosuppressed with a combination of cyclophosphamide and corticosteroids. However, the fungus becomes hypovirulent when the mice are immunosuppressed with corticosteroids alone [69]. In the same way, the deletion of the gene *pld *which encodes Phospholipase D attenuates the virulence in mice immunosupressed with hydrocortisone acetate but not with cyclophosphamide [70]. Phospholipase D modulates the internalization of *A. fumigatus *conidia into host epithelial cells. Phospholipases cleave host phospholipids, resulting in membrane destabilization and host cell penetration [71].

 Fungal amino acid biosynthesis is vital to the pathogen metabolism and a conserved transduction cascade which links the environmental stimuli to amino acid homeostasis is the cross-pathway control (CPC) system. *cpcA* gene in *A. fumigatus* encodes the transcriptional activator of the CPC system of amino acid biosynthesis and the *cpcA *null mutant displayed attenuated virulence in a murine model of pulmonary aspergillosis [72].

 The transcription factor SebA was demonstrated in *A. fumigatus *to be involved in the response to poor nutritional condition, osmotic, oxidative, and heat shock stress. Additionally, in the absence of the gene *sebA,* there is attenuated virulence of *A. fumigatus* in the neutropenic murine model of invasive pulmonary aspergillosis and decreased viability of the fungus during alveolar macrophages phagocytosis [73].

The fungal virulence traits are determined by regulatory elements which control their development and asexual reproduction. MedA is a developmentally regulated protein initially identified in the related model organism *A. nidulans* [74]. The *A. fumigatus medA* null mutant produced conidiophores with impaired phialide and conidia formation, impaired biofilm formation on inorganic substrate, and pulmonary epithelial cell interaction abnormalities such as decreased adherence, damage, and stimulation of cytokine production. MedA is required for normal virulence in an invertebrate and in a murine model of invasive aspergillosis [75]. 

An essential precondition for the beginning and manifestation of an infection is the nutrient uptake availability at the site of the infection. The pathogenic fungi are well adapted to deal with competitors for nutrients and are adapted to the fast environmental shift [76]. *A. fumigatus *is able to uptake nutrients from destruction of the host tissue by its secreted proteases. The main *A. fumigatus *secreted protease is the alkaline serine protease Alp1/asp f 13, which had been shown to be abundant in infected lung tissue and able to degrade some complement components (C3b, C4b, and C5) [77]. 

 In living cells tissue, the iron and zinc availability are in low levels, enough to restrict the growth of pathogens. The transcriptional activator ZafA from *A. fumigatus *regulates the zinc homeostasis and is essential for the pathogenicity and virulence of the fungi, once the *zafA* null mutant is avirulent in a cortisone acetate-immunosuppressed mice [78].

Among micronutrients of the environment, iron is an essential nutrient for *Aspergillus *sp. and a key component of several enzymes that participates in a variety of cellular processes [79]. The iron sequestration is an important factor in host defense against invading fungi, since it prevents *in vivo *fungal development [80]. Low-molecular mass iron-specific chelators called siderophores are employed by the fungus to regulate iron load, which are of great importance in fungal virulence [81]. *A. fumigatus *can acquire iron by two different ways, by reductive iron assimilation and by siderophore-assisted iron uptake. The protein FtrA is a high affinity iron permease which belongs to the reductive mechanism for iron assimilation. The inactivation of *ftrA* gene in *A. fumigatus* does not change the virulence in a murine infection model. By contrast, the low virulence after the inactivation of siderophore-assisted iron uptake components, *sidC*, *sidD*, *sidF,* and *sidG* indicates that siderophore biosynthesis but not reductive iron assimilation mechanism is essential for *A. fumigatus *virulence [82–84]. Among the *Aspergillus* species, the acquisition of iron under depleted condition of this compound is controlled by the proteins SreA and HapX [85, 86]. AcuM is the transcription factor involved in the suppression of *sreA *and induction of *hapX *to stimulate expression of genes involved in both reductive iron assimilation and siderophore-mediated iron uptake, besides gluconeogenesis regulation. *A. fumigatus *Δ*acuM* mutant had reduced iron incorporation, decreased extracellular siderophore production, impaired capacity to grow under iron-limited conditions, and decreased virulence in *Galleria melanogaster *larvae model, as well as in murine models of hematogenously disseminated and invasive pulmonary aspergillosis [87]. Curiously, *sreA *null mutant is as virulent as an *A. fumigatus *wild-type strain, but HapX-deficiency causes significant attenuation of virulence in a murine model of aspergillosis [86]. 

Iron also influences processes such as ergosterol biosynthesis, azole drug resistance, hypoxia adaptation, and the interaction with the host immune cells [88–90]. The iron availability in eukaryotes and cellular response to low oxygen are intimately related [91].

In an infection, *A. fumigatus* is exposed to active changes in the oxygen concentration, once the quantity of oxygen at the site of infection is low due to the inflammatory response. SrbA, related to the sterol regulatory element-binding protein, is critical for coordinating genes involved in iron acquisition and ergosterol biosynthesis under hypoxia and low iron conditions found at the site of the human infection [88]. *A. fumigatus srbA* null mutant is incapable of growth in a hypoxic environment and consequently is ineffective in causing disease in a murine model of invasive pulmonary aspergillosis [92]. Mitochondrial respiration is active in hypoxia and critical for fungal pathogenesis. The deletion of the cytochrome C (*cycA*) which is involved in mitochondrial respiration in *A. fumigatus* led to significant impaired conidia germination, growth in normoxia/hypoxia, and the fungus displayed attenuated virulence in murine model of invasive pulmonary aspergillosis [93]. 

In summary, fungi are versatile organisms able to adapt to diverse environmental conditions in order to grow and survive. In a hostile environment like the human body, *A. fumigatus* must evade intricate host defenses with the help of a growing number of virulence factors that are continuously being described. These molecules, which are expressed and secreted by fungus, enable host colonization, and promote immunoevasion, immunosuppression, and pathogen nutrition. On the other hand, the host immune response status is the key point that defines the progression of a disease, like the pulmonary invasive aspergillosis.

## 3. Host Response to *Aspergillus fumigatus*

Clinical manifestations of aspergillosis are determined by the host's immune response against the fungi and have been classically divided into allergic, saprophytic, and invasive forms [94]. In this context, in atopic individuals, the fungus triggers immune-mediated phenomena such as allergic rhinitis, asthma, and allergic bronchopulmonary aspergillosis (ABPA) [95], involving the upper and lower respiratory tracts. Allergic bronchopulmonary aspergillosis affects 1-2% of asthmatic subjects and 7–9% of cystic fibrosis patients [96, 97]. This pathology is characterized by a T helper 2 (Th2) lymphocyte response, eosinophilia, and increased serum IgE levels [98, 99]. However, in patients with preexisting cavitary lesions as a result of diseases such as tuberculosis, sarcoidosis, bronchiectasis, or cavitary neoplasia [100], growth of the fungus leads to saprophytic aspergilloma [101], which is a saprophytic form of aspergillosis [102]. In immunocompromised individuals, some conidia germinate in the lungs as hyphae, the invasive form of the fungus, causing serious angioinvasive infection, often fatal, known as invasive pulmonary aspergillosis [103, 104].

### 3.1. Innate Immune Response

*Aspergillus* species are moulds widespread in the world, and the most common source of infection is the inhalation of spores (conidia) into the lungs and sinuses [105]. Due to the small size of *A. fumigatus* conidia (3–5 *μ*m) and the presence of a hydrophobic protein-coat layer which underlies immunologically active polysaccharides and consequently protect them from host defense, the spores may penetrate deeply into the respiratory tract where they find appropriate conditions to develop [11, 106, 107]. Although *A. fumigatus* has not been described as an intracellular pathogen, the *in vitro* ability of its conidia to bind to respiratory epithelium cells, to be phagocytosed, and to survive into acid organelles of these cells was already shown [97, 108] suggesting that *A. fumigatus* may use epithelium cells to escape from phagocytes. Besides, the ability of this fungus to cause damage to epithelial cells by reducing the beating of cilia from ciliated cells and thus impairing an important physical barrier which protects host against fungal invasion was already shown in an *ex vivo *model using human cells. This detrimental effect was probably due to the production of mycotoxins such as gliotoxin, fumagillin, and helvolic acid [109]. Other findings showed that respiratory cells may be activated by double-stranded RNA from *A. fumigatus* conidia and initiate an immune response via IFN-*β* production [110].

The recognition of *A. fumigatus* constituents by innate immunity and subsequent signaling is related to pattern recognition receptors (PRRs), which include C-type lectin and toll-like receptor (TLR) families that recognize pathogen-associated molecular patterns (PAMPs) like fungal wall components [111, 112]. As described before, C-type lectin, such as dectin-1, is a receptor for *β*-glucans, one of the fungus wall compounds and one of the major innate receptors for protection against *A. fumigatus. *Dectin-1 deficient mice are more susceptible to fungal infection when compared to control group, as demonstrated in a study where the deficient mice showed an impaired production of inflammatory cytokines and chemokines such as IL-1*β*, TNF-*α*, CCL3, CCL4, and CXCL1, leading to insufficient lung neutrophils recruitment and reactive oxygen species (ROS) production besides uncontrolled growth of *A. fumigatus *in lungs. In this study it was also observed an impaired capability of macrophages to produce proinflammatory mediators in response to fungus infection in the absence of dectin-1 [112]. This receptor, which is also a structural component of the corneal epithelial cells, has its expression increased early after *Aspergillus fumigatus* infection of the cornea of rats [113]. 

The role of TLR family in the host defense against *A. fumigatus* infection was already described too. These receptors are able to activate the antifungal properties of many innate leukocytes such as macrophages and polymorphonuclear (PMN) cells. Netea et al. demonstrated that TLR2 is related to the recognition of yet unidentified ligands of conidia and hyphae, whereas TLR4 was only associated with the recognition of conidia ligands [114]. The activation of TLR2 by *A. fumigatus *was observed with the formation of the heterodimers TLR2/6 in mice and TLR2/1 both in human and mice cells *in vitro* [115]. The activation of TLR4 led to the oxidative pathways with azurophil and myeloperoxidase granules release [116]. Rubino et al. using an *A. fumigatus *strain (Δ*rodA* CBS 144-89) deficient in a protein (Rod A), which prevents innate immune recognition, demonstrated both in human and murine cells the role of TLR2 and TLR4 in the recognition of this fungus by innate immune cells beyond the dependence of TLR1 (in human and mice cells) and TLR6 (in murine cells) for recognition [115]. Therefore, to evaluate the role of TLR in the response against *A. fumigatus, *bone marrow-derived macrophages from wild-type, TLR1, TLR2, TLR3, TLR4, and TLR6 knockout mice, were coincubated with the fungus. A reduced production of proinflammatory cytokines and chemokines such as IL-12p40, CXCL2, IL-6, and TNF-*α* from TLR1 deficient macrophages was observed, and this production was almost abolished in TLR2, TLR4, and TLR6 knockout cells but not in wild-type or TLR3 deficient macrophages, showing the importance of TLR1, TLR2, TLR4 and TLR6 in the fungal recognition and clearance. Moreover, to evaluate the role of TLR in human cells, Rubino et al. also used HEK293T cells transiently transfected with vectors of human TLR1, TLR2, or TLR6 and demonstrated that only in the presence of human TLR1 and TLR2 *A. fumigatus* was recognized, and there was activation of the inflammatory transcription factor NF-*κ*B. Furthermore, the importance of TLR6 was reinforced in a study conducted with mice deficient in this receptor, in a model of *A. fumigatus* allergic induced asthma. In this work it was observed that in the absence of TLR6, there was a lower production of IL-23 and Th17 response, which resulted in exacerbated asthma response [117]. Regarding TLR4, when this receptor (and coreceptor CD14) was blocked with specific monoclonal antibodies in human monocytes* in vitro* (using the concentrations of 1, 3, or 10 *μ*g/mL), an inhibition between 35–70% of TNF-*α* releasing was observed especially when the highest concentration was used, thus highlighting its relevance on innate immune recognition of *A. fumigatus* [118]. On the other hand, triggering TLR2 induced oxidative pathways in PMN with the release of gelatinases and inflammatory cytokines [116]. To note, gelatinases that are extracellular matrix destructive enzymes [119] are associated with PMN migration during inflammation and are mobilized more readily than other molecules such as lactoferrin and azurophil granules [120]. In ABPA, caused by *A. fumigatus*, an increased inflammatory infiltrate containing neutrophils is found as well as elevated levels of the gelatinase MMP-9 and the cytokine IL-8, compared to asthma patients and controls. Moreover, IL-8 correlated with neutrophils and MMP-9 in the sputum, suggesting that the influx of neutrophils modulated by this cytokine may lead to a putative MMP-9-mediated tissue damage in the lungs [121]. Moreover, triggering of TLR3 were attributed to *A. fumigatus *conidia double-stranded RNA [110, 122], leading to contrasting results when compared to the study of Rubino et al. [115]. The opposite responses generated by TLR3 activation throughout the different studies were probably due to the strains used in them, besides different cellular and activation contexts. Therefore, such divergent results may be the consequence of diverse experimental conditions used by the authors of the studies. In addition, TLR9 activation was related to unmethylated CpG motifs present on the fungus DNA [123]. Thus, the importance of TLR seems clear, especially, TLR1, TLR2, TLR3, TLR4, TLR6, TLR9, and C-type lectin such as dectin-1 on *A. fumigatus* recognition by innate immune cells. Furthermore, MyD88, which is a molecule that mediates signaling downstream activation of most of TLRs mediates fungal clearance, inflammation, and tissue injury early after pulmonary infection with *A. fumigatus* [124]. 

Nucleotide-binding oligomerization domain (NOD) proteins (NOD1 e NOD2) are a subset of PRRs that recognize intracellular pathogens containing molecular patterns such as peptidoglycan. Therefore, NOD2 recognizes bacterial muramyl dipeptide (MDP) both from gram-positive and gram-negative bacteria [125], and its importance on immune response in different organs such as intestine [126] and lungs [127] was also observed. After the peptidoglycan detection the NOD proteins recruit the downstream adaptor molecule RIP2 hence activating proinflammatory pathways such as NF-*κ*B and mitogen-activated protein (MAP) kinases such as p38, ERK, and JNK [128]. NOD2 is also expressed by a human corneal epithelial cell line, especially after activation with *A. fumigatus* conidia, which then triggers the production of proinflammatory cytokines such as TNF-*α* and IL-8 through the NF-*κ*B pathway [129]. Similarly, the stimulation of a macrophage cell line with *A. fumigatus* conidia resulted in significantly increased expression of NOD2, RIP2 and NF-*κ*B with the production of proinflammatory cytokines in a NOD2-dependent manner [130]. Therefore, this initial innate detection of *A. fumigatus* triggers important events for fungal control and clearance such as the production of inflammatory mediators, recruitment, and activation of immune cells. 

**Figure 1:**
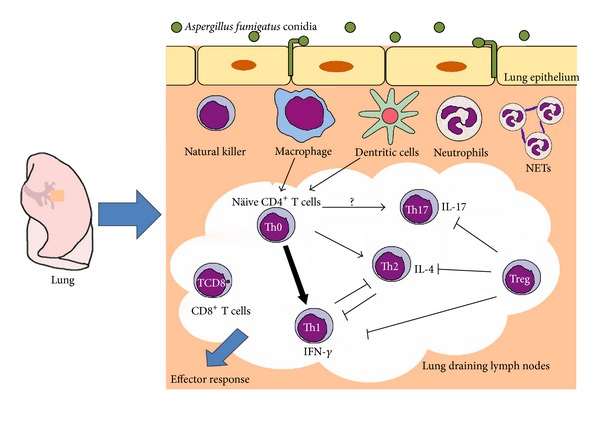
Summary of *Aspergillus fumigatus* and host immune system interplay. The lung pulmonary epithelium is magnified, and the immune response against fungal invasion is demonstrated. First, fungal components are recognized through pathogen recognition receptors (PRRs) which led to the activation of the innate immune response, depicted by macrophages, dendritic cells, natural killer cells, and neutrophils. The role of neutrophils during fungal infection is also represented by formation of neutrophils extracellular traps (NETs). The innate immunity triggers the development of an acquired immune response in the lung draining lymph nodes, which may induce the differentiation of Th1, Th2, or Th17 cell phenotypes depending on the specific stimuli and cytokine milieu, which also accounts with the presence of cytotoxic CD8 T cells against the fungus. These effector antifungal responses may also be modulated by the action of regulatory T cells (Tregs) to avoid excessive tissue damage.

The recognition of fungal cell wall compounds through PRRs is the beginning of the immune response, and the first components of defense against *Aspergillus *spp. in the alveoli are the alveolar macrophages (Figure 1), which phagocyte dormant or swollen spores, killing only swollen spores, especially due to the capability of lung resident macrophages to produce ROS [131]. Accordingly, Ibrahim-Granet et al., using murine and human alveolar macrophages, demonstrated that the activity of phosphatidylinositol (PI) 3-kinase during the initial steps of phagocytosis is required to properly kill spores in addition to the acidification of phagolysosome [132]. 

Although macrophages were described for many years as the main responsible for *A. fumigatus* clearance [133], human and murine neutrophils (Figure 1) also seem to be essential to a successful response against this fungus, due to their ability to form NETs (neutrophil extracellular traps) or inhibit the growth and formation of both spores and hyphae [134, 135]. This fungal control by NETs occurs by the ability of neutrophils to reduce the polar germ tube growth coupled to the presence of calprotectin which may mediate the chelation of Zn^+2^ ions. Moreover, the importance of formation of NETs to control fungal infection was reinforced in different studies using cells from patients with chronic granulomatous disease, which is a rare inherited disorder where neutrophils have a defect on ROS production, thus leading to increased susceptibility to bacterial or fungal infections [136, 137]. To note, the formation of NETs was first described in bacterial infection, as a property of innate immune cells to prevent microorganisms spreading due to a high local concentration of DNA coupled to a set of cytoplasmatic and granular proteins such as histones, elastase, and calprotectin, thus enhancing the capability of immune system to control infections [138–140]. To reinforce the importance of neutrophils in the control of *A. fumigatus,* Mircescu et al. observed, after depletion of alveolar macrophages or neutrophils, that mice infected with *A. fumigatus* had a greater presence of invasive fungus hyphae only when neutrophils were depleted when compared to the macrophages depleted group [141]. Despite the formation of NETs, neutrophils may also act against *A. fumigatus* infection through the production of ROS, as described by Sugui et al. who observed an up-regulation of catalases, superoxide dismutase, and thioredoxin reductase in the phagolysosome in an *ex vivo* model using human neutrophils [142]. Moreover, fungus phagocytosis by neutrophils led to a shift on fungus metabolism resembling a state observed under glucose limitation, suggesting that neutrophils may create nutrient limiting conditions to facilitate fungal killing [142].

Despite the importance of macrophages and neutrophils as the first barrier to control *A. fumigatus* infection, the role of other innate immune cells cannot be discarded. The ability of monocytes to phagocyte and inhibit the spores germination was already showed using human cells. The different monocyte subsets CD14^+^CD16^−^ and CD14^+^CD16^+^ react distinctly to *A. fumigatus* conidia either by controlling germination and secreting low levels of TNF or by producing high levels of inflammatory cytokines while not being able to suppress germination of conidia, respectively. These data indicate that the monocyte subsets may differ in their response to *A. fumigatus* infection depending on the cell phenotype that encounters the pathogen [143]. During experimental lung infection with *A. fumigatus*, inflammatory monocytes can differentiate into monocyte-derived dendritic cells and transport spores to lung-draining lymph nodes, thus making a link to acquired immunity and expansion of CD4-specific T cells [144]. Moreover, a differential regulation of expression of 1.827 genes was observed *in vitro *in human monocytes after their coincubation with *A. fumigatus* conidia [145]. Among these genes those related to host defense against fungal infection were upregulated between 2 and 6 hours of exposure to the fungus, such as IL-1*β*, IL-8, CXCL2, CCL4, CCL3, and CCL20, coinciding with an increase in phagocytosis. Another study, using the same model of coincubation, showed a differential regulation of 602 genes with an upregulated expression of IL-8, CCL20, and CCL2 on monocytes after 3 hours of coincubation with *A. fumigatus* hyphae, in contrast to only 206 genes in response to resting conidia [146]. 

The importance of natural killer (NK) cells to control *A. fumigatus* infection was first described in neutropenic mice that received adoptive transfer of NK cells [147]. The authors observed a reduction in CCL2 expression in the lungs early during fungal infection, and when this protein was neutralized a greater mortality and fungal burden in lungs were observed. This neutralization was also accompanied by a reduction in NK cells recruitment into the lungs, suggesting an important role of these cells in the control of *A. fumigatus* infection. Similarly, early during *A. fumigatus* infection in neutropenic mice, NK cells were the main source of IFN-*γ* production, and when these cells were depleted a reduction in the level of this cytokine was observed, coupled to a higher fungal load in the lungs [148]. In addition, when the authors used adoptive transfer of NK cells from wild-type, but not from IFN-*γ* deficient mice, there was a greater fungal clearance from the lungs, indicating that NK cells function as a relevant source of IFN-*γ* for this fungal control (Figure 1). Furthermore, the importance of NK cells was also related to its *in vitro* capability to damage *A. fumigatus* hyphae, but not resting spores, especially when incubated with alveolar macrophages, resulting in 2–8-fold greater killing and a marked induction of CXCL9, CXCL10, and CXCL11 when compared to macrophages alone or macrophages incubated with IFN-*γ*-deficient NK cells [148]. NK cells may also damage *A. fumigatus *by directly releasing soluble factors such as IFN-*γ* [149]. Taken together, these findings indicate that complex and multifactorial innate processes may act as the initial defense mechanisms to avoid *A. fumigatus* lung infection and disease establishment. 

### 3.2. Acquired Immune Response

Innate immunity alone is able to control fungal infections especially at low doses; however, when the airway infections become frequent or high fungal burdens occur, acquired immunity is necessary to host protection [150]. Accordingly, the role of CD4 (including Th1, Th2, Th17 and regulatory) or CD8 T-cell responses during *A. fumigatus* spore exposure was already showed in experimental (mice) and *in vitro* assays using human cells. In these studies different T helper cell responses were observed depending on the fungal specific components to which T lymphocytes were exposed, thus inducing protective or harmful reactions; that is, fungal secreted proteins activated nonprotective (Th2) IL-4 secreting clones, glycolipids led to nonprotective (Th17) IL-17 activated response, and polysaccharides induced protective IFN*γ*, IL-17, or IL-10-clones besides GPI-anchored proteins (Gel1p and Crf1p) and proteases (Pep1p) leading to Th1/Treg protective reactions [151]. Therefore, Th1/Treg response plays a protective/regulatory role, whereas Th17 could have a protective or a harmful role depending on which fungal specific component this T helper cell subtype interacts with. However, Th2 cells seem to play a nonprotective role during *A. fumigatus* infection and are especially activated after interaction with fungal secreted proteins. 

The predominance and contribution of Th2 cells (Figure 1) to disease progression were also observed in cystic fibrosis patients who develop ABPA [152, 153]. To note, individuals with cystic fibrosis present an overall risk of 4–15% to develop ABPA [153]. In fact, the dendritic cells of cystic fibrosis patients with ABPA present elevated expression of the costimulatory molecule OX40 ligand coupled to a lower secretion of TGF-*β*, a profile linked to a Th2-biased cell response which is not observed in cystic fibrosis patients without ABPA [152]. In an experimental murine model with repeated challenges using *A. fumigatus* conidia, especially after four exposures, there was a predominance of IgE, eosinophils, and IL-4, all are hallmarks of Th2 response [154], thus suggesting that the Th2 response predisposes to *A. fumigatus *infection. Moreover, Shreiner et al. demonstrated the importance of IL-4 and especially IL-10 in the generation of a Th2 response during an 8-week experimental chronic infection with this fungus, rather than acting as anti-inflammatory cytokine [155].

On the other hand, the protective role of Th1 response (Figure 1) during *A. fumigatus* infection was reinforced both in immunocompromised patients [156, 157] and in neutropenic mice due to its capability to neutralize Th2 cytokines [158]. In this context, Cenci et al. showed that when CD4 T cells produced IFN-*γ in vitro* and not IL-4, a protective acquired immunity was developed. This evidence was also clear when the animals infected with *A. fumigatus *were treated with IFN-*γ* and a lower mortality rate was observed when compared to the nontreated group. The importance of monocyte-derived dendritic cells to drive CD4 T cell differentiation during *A. fumigatus *infection was also observed. In the absence of dendritic cells there is a prevalence of Th17 over Th1 phenotype, together with the lost of T-bet expression in CD4 specific T cells, suggesting that monocyte-derived dendritic cells are important for the maintenance and development of protective Th1 cell response against the fungus. In addition, the predominance of Th1 response against *A. fumigatus* was also demonstrated in healthy individuals [159]. 

For many years, until the discovery of Th17 cell subtype [160], the occurrence and immunopathogenesis of fungal infections was attributed only for the traditional balance/imbalance between Th1 and Th2 responses [161–163]. However, in the last years different studies have shown a controversy role for Th17 response (Figure 1) during *A. fumigatus* infection. In mice experimentally infected with *A. fumigatus *or *Candida albicans*, the production of Th17 signatures cytokines, such as IL-23 and IL-17, may play a nonprotective role during fungal infection due to its ability to negatively regulate the development of Th1 response and to impair the *in vitro *neutrophil-mediated killing or the *in vivo* fungal clearance [164]. To reinforce the negative influence of Th17 cells on fungal control, these authors blocked *in vivo *the IL-23 and IL-17 cytokines and observed an increased resistance to both infections, as evaluated by the observation of a decreased fungal growth in the lungs. Despite the permissive role of Th2 response in the progression of *A. fumigatus* infection, the deleterious participation of Th17 cells was also observed in an experimental model of chronic infection in which the inflammatory reaction was characterized by eosinophilia and Th2 cell-associated cytokine profile coupled to IL-17 production, which persisted even after Th2 response, had begun to resolve [165]. These data indicated that Th17 could be as detrimental as Th2-biased response for host during *A. fumigatus* infection. In this study, after repeated challenges with fungus conidia, when an IL-17 knockout mice was used, the inflammation was attenuated and fungus clearance was enhanced. On the other hand, the protective role of IL-17 in the host defense against this fungus was also observed in another experimental model of *A. fumigatus* infection. In this case, the *in vivo *neutralization of this Th17-related cytokine early during infection resulted in an impaired pathogen clearance that led to an increased fungal pulmonary burden [112]. Therefore, the exact role of IL-17 in the *A. fumigatus* infection is still not totally clear yet and may depend on the host, time of infection, or even contact with fungal specific wall components [151].

The expansion of regulatory T cells (Tregs) (Figure 1) during fungal infections may constitute an important tool to avoid deleterious or exacerbated inflammation due to Th1 response or hypersensitivity reactions associated with Th2 responses [154]. Tregs may play a relevant role early in the *A. fumigatus* infection by modulating proinflammatory activities of polymorphonuclear leukocytes through contact-dependent or independent mechanisms such as IL-10 production [166]. Otherwise, later during *A. fumigatus* infection Tregs may work especially by inhibiting Th2 cell response and then preventing allergy to the fungus. 

Besides CD4 T helper responses, it is know that CD8^+^ T cells may also mediate protective immunity against *A. fumigatus* infection, especially through recognition of HLA-A*0201 restricted fungal peptides [167]. Furthermore, dendritic cells may recognize fungal RNA by TLR3 and induce class I MHC restricted CD8 T-cell protective responses to *A. fumigatus* [122]. In addition, Templeton et al. observed increased IFN-*γ* producing CD8^+^ T cells in bronchoalveolar lavage fluid of mice repeatedly challenged with *A. fumigatus *conidia coupled to the maintenance of airway memory phenotype CD8^+^ T cells [168]. The maintenance of this CD8^+^ T cells in airways could be attributed to the specific characteristic of *A. fumigatus* conidia to germinate and persist in lungs, thus pointing to cytotoxic cells as another important mechanism for fungal control.

## 4. Concluding Remarks

In summary, this review showed that the control of *A. fumigatus *infection is associated with different and interconnected mechanisms dependent on the interplay between the pathogen virulence factors and the host immune competence. Recently it was showed that the immune response induced by *Aspergillus* spp. may be dependent on variations of the fungus strain that could present diverse virulence factors and therefore increased or reduced infectivity [169]. Even though, in general, the infection control may begin early during pathogen invasion when the fungus crosses physical or chemical natural barriers which, if disrupted, may lead to the pathogen recognition and activation of innate immunity receptors and cells like macrophages and neutrophils that, in turn, may initiate the acquired immunity events represented by CD4 or CD8 T-cell activations. In this context, a consensus among the authors that Th1-biased response is protective seems to exist, due to its ability to recruit other cells and restrain fungal growth, while the Th2 cell response is harmful especially to be associated with higher fungal burden at lungs or the occurrence of allergy. In addition, a controversy remains regarding the role of Th17 responses during *A. fumigatus *infection, while the cytotoxic activity of CD8 lymphocytes may be essential in some circumstances of intracellular pathogen. Overall, the regulation of leukocyte excess inflammation and avoidance of tissue damage directed to the elimination of *A. fumigatus *is related to Tregs activity. However, further studies must be conducted in order to better clarify how certain fungal compounds may activate or escape from the immune system as well as to provide tools for the development of novel therapeutic approaches to control this fungal lung infection.
